# Why Breast Cancer Patients Seek Traditional Healers

**DOI:** 10.1155/2012/689168

**Published:** 2011-11-17

**Authors:** Mazanah Muhamad, Sharan Merriam, Norhasmilia Suhami

**Affiliations:** ^1^Cancer Resource and Education Centre, Universiti Putra Malaysia, 43400 Serdang, Selangor, Malaysia; ^2^Department of Lifelong Education, Administration and Policy, College of Education, University of Georgia, 850 College Station Road, Athens, GA 30602, USA; ^3^Institute for Social Science Studies, Universiti Putra Malaysia, 43400 Serdang, Selangor, Malaysia

## Abstract

Traditional healing is a common practice in low and middle income countries such as Malaysia. Eighty percent of Malaysians consult traditional healers or “bomoh” at some time in their life for health-related issues. The purpose of our study was to explore why breast cancer patients visit traditional healers. This is a qualitative study utilizing in-depth interviews with 11 cancer survivors who sought both traditional and Western medicine. The findings revealed the following reasons for which patients seek traditional healers: (1) recommendation from family and friends, (2) sanction from family, (3) perceived benefit and compatibility, (4) healer credibility, and (5) reservation with Western medicine and system delay. These factors work together and are strongly influenced by the Malaysian cultural context. The issue with the Western health system is common in a developing country with limited health facilities.

## 1. Introduction

Traditional healers are an important component of Malaysian healthcare. The Malay traditional healer is locally referred to as “bomoh.” It is estimated that upwards of 80% of Malaysians consult bomohs at some time in their life for health-related issues [1]. In the Malaysian context, it is not known how many bomoh there are because they are not recognized by the government and are not officially registered. However, they are numerous, especially in states like Kelantan where 90% of the population is Malay [1]. Bomohs are so embedded in the culture that they are sometimes the first and only source of information and treatment. Even those who seek standard Western medical treatment often simultaneously consult bomohs. One of the issues with cancer is that by first seeing a bomoh, diagnosis and treatment are delayed resulting in an unusually high rate of diagnosis of advanced stages of cancer, especially in Malay patients compared to Chinese and Indian ethnic groups [2].

Malaysia is a country of 26 million people in Southeast Asia. Peninsular Malaysia is bordered on the North by Thailand and on the South by Singapore. The two states of Sabah and Sarawak on the Island of Borneo constitute what is known as East Malaysia. It is a multicultural society consisting of approximately 60% Malays, 30% Chinese, 10% Indian and others. While it is difficult to get an accurate assessment of the prevalence and morbidity rates of cancer in Malaysia, it is widely considered to be on the rise. The National Cancer Registry (NCR) has been in existence only since 2002 and reports are based on sometimes erratic hospital epidemiology data [3]. Further, the NCR has statistics from Peninsular Malaysia only (statistics from East Malaysia are not available).

From the data that are available, there were 21,773 new cancer cases diagnosed in 2006 in Peninsular Malaysia. Malaysian cancer statistics from the National Cancer Registry [4] reveal that cancer incidence is higher among females. The five most common types of cancer among all Malaysians are breast, colorectal, lung, cervix, and nasopharynx. The most frequent type of cancer in males is colorectal, while the incidence of breast cancer in women is much greater than other types of cancer.

Breast cancer is the most common cancer in women in Malaysia. Compared to the West, breast cancer occurs in younger women in the country, tumor mass at presentation is larger [5] and “30–40% present with late breast cancer (Stage 3-4) compared to Western countries where more than 80% of women present with early stages” [3]. With regard to the three races in Malaysia, 40% of Malays present with Stage 3 and 4, while only 15% of Chinese and 20% of Indians present with Stage 3 and 4. Further, Malay women have the poorest survival rate, “since they tend to present with later stages” [3]. According to Hisham and Yip [6], the popular practice of traditional healing in less developed and developing countries is associated with late presentation.

Cancer awareness, diagnosis, and treatment in Malaysia are hampered by lack of resources and trained personnel. For example, “the total number of oncologists in Malaysia is 35,” resulting in an “oncologist: population ratio of 1 : 650,000” (the UK ratio is 1 : 250,000) [3]. Lack of cancer treatment facilities, radiotherapy machines, and so on result in restricted access to treatment, and if access is facilitated, there is often an extended waiting time for treatment [3].

Complementary and alternative medicine (CAM) refers to the field of health care outside or beyond the traditional, allopathic, or Western biomedical medicine [7]. Complementary and alternative medicine is used widely and reflects many needs and concerns that are not being met by conventional medical practices. Moreover, CAM poses new challenges to modern oncology. Although there is no evidence that alternative medicine is more effective than conventional medicine, public opinion and interest in CAM is strong and growing [8]. According to Morris et al. [9], breast cancer patients tend to use CAM more compared with other cancer patients. As a multiethnic, multicultural, and multireligious country, Malaysia is in a unique position as it serves as a confluence of three Asian cultures giving rise to three healing traditions of the Malay, Chinese, and the Indian. Although at present the main health care delivery is through conventional (Western) medicine, there is undoubtedly a growing interest in traditional and complimentary medicine (TCM) in the country. The Malaysia's National Health and Morbidity Survey II (1996) reported that 2.3% of the population visited TCM practitioners, and 3.8%, visited both modern and TCM practitioners [10]. The interest led to the legislation of TCM in 2001 [11] and the implementation of TCM in four public hospitals in Malaysia [12]. The variety of traditional health care systems reflecting the various ethnic groups coexist with modern health care systems and are widely used by Malaysian communities [13].

One of the popular TCM practices in Malaysia is traditional healing. The first National Health and Morbidity Survey in 1986 recorded that Malaysians made 0.1 visits per person per year to traditional healers as compared to 1.4 visits per person per year to out-patient clinics [8]. One study found that ninety-six percent of mental health patients in Malaysia had visited a traditional healer before seeking psychiatric care [14]. In terms of cost, WHO estimates that “in Malaysia, an estimated US $500 million is spent annually on this type of health care, compared to about US $300 million on allopathic medicine” [15].

This inquiry focuses on *Malay* traditional healers (note that Chinese and Indian healers are not part of this study). There are several types of Malay traditional healers including the following: (1) Islamic healers who draw on Islamic religious beliefs and use verses from the Quran as the major component of their treatment; (2) traditional healers who “know the folklore of disease causation, treatment and prevention” and are known by the term “bomoh.” Bomoh uses a variety of handed-down, traditional methods in diagnosing and treating patients including “herbal remedies, ceremonial rites, incantation, exorcism and sorcery” [14], (3) “bomoh patah” which loosely translates to bone doctors or bone setters [16]. There is some overlap among these types, however. An Islamic healer may also use herbs and other traditional methods, and a traditional bomoh may use some Islamic prayers and may also treat bone fractures. These three types of healers can be found throughout Malaysia. Why do breast cancer patients in Malaysia seek traditional healers? To date, there is no specific study that inquires into the issue. The closest was conducted by Taib et al. [17], who studied 25 women who presented with locally advanced and/or metastatic cancer. Sixty percent of the women studied used some form of alternative therapy including oral preparations, applications and spiritual prayer. Since this paper deliberates on motives why patients seek traditional healers, the following section will review what is known about the motives.

### 1.1. Previous Research

From reviewing the literature, we found some research related to this study from 10 different countries. As mentioned above, the closest study to our context was done by Taib et al. [17]. As revealed by Taib et al. [17], there are a number of possible motivations for seeking therapies outside the Western medical system, including fear of surgery, a friend's influence, an earlier bad experience in a hospital, thought that alternative therapy works, financial problems, fear they would be unable to work after surgery, thought that it was not cancer, and shy about seeing a doctor. Taib et al. [17] also found that 15.5% of cancer patients sought alternative treatment *before* visiting a breast cancer clinic. In developing countries, inadequate medical facilities in the primary health system, poor infrastructure, and information systems become a barrier to the development of comprehensive screening programs and the effective control of cancer [18]. Moreover, lack of financial resources often compels many cancer patients to venture into inexpensive alternative medicines in search of cure and palliation [18–20]. A previously unpleasant experience in a hospital also drives cancer patients to seek traditional healers [17]. Misconception about Western healing is another push factor towards traditional healing. Leong et al. [21] reported that patients's fear of surgery will disturb the cancer and cause it to grow larger. Overall, the inadequacy or failure of modern medicine and the health care system lead patients to turn towards traditional healing [22].

In addition, one's culture, belief system, and interpretations of the meaning of illness have important effects on what approaches to treatment are sought when illness strikes [23]. Struthers and Eschiti [23] adds that “When confronted with a life threatening illness such as cancer, an option for people who are familiar with, comfortable with, and believe in traditional healing is to integrate Western medicine with traditional healing.” Leong et al. [21] stated that a strong traditional influence causes women to initially seek traditional or alternative treatment, such as “bomoh” or faith healing before they present to a hospital. Al-Adawi [20] points out that the therapeutic techniques congruent with the local beliefs and traditions are reasons for seeking traditional healers. As sickness was shared by the community, seeking a traditional healer increases the patient's self-value and reinforces the patient's relationship with the community [20]. Al-Adawi [20] contends that in many traditional communities, the role of healer in health services has remained central. Traditional healing practices are not limited to traditional communities. According to Malik et al. [24] even in Western countries, 6 to 45% of cancer patients used unconventional therapies. The practice is also influenced by cultural values, religious beliefs, language of clients, and social trends [24].

The role of culture in influencing traditional healing was also reported by research in Canada [22], Indonesia [25], and Malaysia [26]. Moodley et al. [22] argue that traditional healers are familiar with their client's behavior, belief, values, and language. Salan and Maretzki [25] who inquired into mental health and traditional healing in Indonesia, further add that traditional healers are able to view illness from the patient's cultural perspective. The healer's advice was accepted as it was brought up within the patient's cultural ambience and values [25].

Traditional healers give people hope and treat patients as someone who can be cured. According to Lau [27], this sense of hope and the prospect of help contribute towards the patient's improvement. Mohamed et al. [28] added besides hopefulness, seeing a healer may also give a sense of control to the individual. Further, traditional healers tend to offer patients and their families a more personal and intimate relationship that most trained doctors and hospital staff cannot offer [27]. From Heggenhougen's [16] viewpoint, healers encourage positive feelings and faith that have a curative value for physiological and physical ailments. Other traditional healers' attractions are understanding a patient's background and providing advice, solace, and therapy [1].

Tovey et al. [29] argue that traditional medicine may play a more crucial role in the patient's emotional and spiritual well-being than potentially curative options. This spiritual healing role is supported by studies in countries such as Norway, where faith healing is the commonest method practiced by users of unconventional therapy [30]. Similarly in Pakistan, spiritual therapy was commonly practiced [24].

According to Al-Adawi [20] healers' accessibility and respected status are their main attractions. An early study by Heggenhougen [16], showed that 28% of patients reported their confidence in the healer, because previously a problem had been cured or they knew others who had been cured. Ninety-two percent of patients reported they felt better after seeing the healer, because the healer instilled confidence that the problem could be solved [16]. Further, the healer was a religious man. Thus patients believed he was a medium for God's healing. Razali and Yassin's [1] study of Malaysian psychotic and epileptic patients who sought complimentary healing revealed that the belief that patients could be cured was among the motivating factors.

In their attempt to cope with their health issue, patients often resort to both traditional and allopathic medicine [29]. Cancer patients in the United States used unconventional methods either as an adjunct to conventional therapy, or after the conventional therapies had been exhausted [31]. A patient may perceive that an illness may be dealt with from their cultural tradition. Thus patients consulting both the Western health worker and traditional healer do it for different reasons; the Western one is for curative treatment while the traditional healer is for cultural explanations [32].

Friends and family play a crucial role in influencing patients to seek a traditional healer. This is evidenced in Razali and Yassin [1] study in Malaysia, and in Pakistan, 83.7% of cancer patients were influenced by their family members to use these methods [19]. Thirty-six percent of cancer patients use these either before receiving any conventional therapy, or after becoming tired of trying conventional therapies [19]. Hasan et al. [12] also observed that besides health professionals and advertisement, friends and family members were the main sources of influencing a patient with chronic disease to use CAM. 

In Tovey et al.'s study of cancer care in Pakistan, unconventional methods were perceived as useful and non-toxic by patients and were often used prior to presentation to physicians [19]. Moreover, according to Al-Adawi [20], traditional medicine is able to give a comprehensive health care service which takes care of the patient's physical, social, spiritual, and psychological aspects [20]. Freeman and Motsei [32] explain that traditional healing involves the whole person which means that care is directed to the biological aspect of the person and also at the social, psychological, and spiritual as well.

As reported above, what is known about motives for seeking traditional healers comes from studies on patients with various diseases in various places such as Canada [22, 33], Indonesia [25], India [18], Norway [30], Pakistan [19, 24, 29] and USA [7, 9, 23, 28, 31, 34, 35]. These inquiries give us a glimpse into what might be going on, but to date there are no studies specifically with breast cancer patients in Malaysia who seek traditional healers. Due to the fact that breast cancer is the main cancer in the country [4], that Malay women have the poorest survival rate compared to other ethnic groups [2], and that this poor survival rate is due in part to late presentation [3], and that the late presentation is associated with their consultation with traditional healers [5, 36], it was important to know why the Malays diagnosed with breast cancer visit traditional healers. Hence, the purpose of this study was to understand what motivates cancer patients to seek traditional healers. Since little is known why Malay cancer patients seek traditional healers prior to and during their diagnosis and treatment, a qualitative approach was deemed the most appropriate design and data collection method [37, 38].

## 2. Methodology

### 2.1. Data Collection

Although cancer patients do seek traditional healers, many remain anonymous. Thus for this inquiry, participants were identified by referrals from support group members, friends, and relatives of cancer survivors, and recommendation by the traditional healers themselves. We sought cancer survivors who had accessed both the Western medical system and traditional healers in the course of their diagnosis and treatment. We developed the interview guide for the in-depth interviews based on the research question, literature, and experience of one of the researchers who is a local, a cancer support member, and a survivor herself.

From the snowballing technique engaged in getting the informants, we interviewed a total of 11 breast cancer survivors. Five of the 11 are from Kuala Lumpur (the capital city which is centrally located) and surroundings. The rest are from Northeast, Northwest and Southern Peninsular Malaysia. The interviews were conducted at a place of the patients' choosing and five chose their home. Three interviews were done at cancer support centres, two at the informant's offices, and one at a restaurant. All interviews were tape-recorded and later transcribed verbatim. Four of the 11 interviews were conducted in English. The transcribed interviews in Bahasa, Malaysia were translated into English by translators of Malaysian Translation Association and by bilingual graduate students. All translated interview transcripts were double checked for accuracy by the local researcher.

### 2.2. Data Analysis

Verbatim transcription of the interviews formed the database for the analysis. Interview data were analyzed using the constant comparative method of data analysis [37–39]. This method consists of first open-coding each interview transcript for relevant data responsive to the study's research questions. These coded segments are then combined through axial coding into themes/categories that are explanatory of the phenomenon. The same process is undertaken with the transcript of the second interview. Themes/categories from the second transcript are compared with the first transcript and one set of themes/categories is derived from the two interviews. This process continues through subsequent interviews. The final sets of themes/categories are the findings of the study. These findings are in turn supported by quotes from the transcripts. These quotes are the evidence for the findings.

It should be noted that in qualitative research, data analysis is best done concurrently with data collection. We informally debriefed after each interview and informally compared respondents' perspectives as we were collecting data. Concurrent data analysis allows for theoretical sampling to take place (adjusting who we want to interview according to our emerging findings) and allows for checking for internal validity (whether we are representing participants' perspectives and understandings as we check our emerging understanding with participants). We concluded data collection after the eleven interviews as the data were saturated; that is, no new insights were forthcoming in the final interviews.

### 2.3. Ethical Consideration

This inquiry was carried out as part of the university's research agenda. Thus, the institution granted the ethical clearance. Prior to the interview, participants were informed of the research objective and the interview process, and assured of their anonymity and the confidentiality of their responses. Pseudonyms are used in this paper. Written consent was obtained from participating informants. Participants were presented with a certificate of appreciation acknowledging their participation.

### 2.4. Study Limitation

In qualitative research generalizability is not possible in the statistical sense. Rather, qualitative research is designed to understand and describe the phenomenon from the perspective of the participants. The findings of a qualitative study are supported by the evidence gleaned from the interviews and it is up to the reader or consumer of the study to determine the applicability of the findings to their situation.

## 3. Findings

Data were obtained from 11 breast cancer survivors as can be seen in Table 1.

The women have survived the disease ranging from 2 to 12 years. The age range was 34–75 years old. Their level of education varied from 6th grade to Ph.D., with the majority being high school graduates. Most of the women were employed. Various factors motivated informants to seek traditional healing in addition to Western medicine. The motives can be grouped into five strands: (1) recommendation from family and friends, (2) sanction from family, (3) perceived benefit and compatibility, (4) healer credibility, and (5) reservation with Western medicine and system delay. The following details the motives.

### 3.1. Recommendation by Family and Friends

Five informants reported that seeing a traditional healer was recommended by their family, and another five said friends persuaded them. In Norzaini's case, it was her father: “Whenever they (the family) heard there is a healer, my father said “go and try.” The recommendation also came from her brother.

For Rashidah, a businesswoman, her friends recommended that she go and see an Islamic healer. Some friends were very insistent. For example, Aira's friend even offered to take her to see the healer: “My friend said, “you have to take leave (from work), my husband and I will take you to this healer, a bomoh in Pahang” (a State quite distant from where Aira and her friend live).

A cancer patient may not believe in traditional healing. Despite the reservation, he/she may end up in seeing one upon recommendation from family or friends. For example, Robiah, a homemaker, did it upon her sister's advice: “But for me, I do not believe (in traditional healing). But she was my sister (who made the recommendation), I just take it” (the suggestion).

### 3.2. Sanction from Family

Family roles in motivating survivors to see a traditional healer goes beyond recommending. The informants reported that family sanction matters. Nowan went to see the healer first (before a doctor), according to her family's wish: “My family agreed that I should see the bomoh first, then the doctor.” Aira, an educated married woman went to see the healer with her father's permission: “He said that whatever it is you have to go for treatment, of course go to the hospital. But he has no objection for me to see the bomoh.” For Aisan, her father in-law's blessing was essential: “People recommend me to see traditional healer. But when my father-in-law said, “Ok, there is someone good at it,” I just went.” Besides parents, spouse sanction is also crucial. According to Aisan, her husband read about the side effects of conventional treatment and encouraged her to go to a traditional healer instead. “He supports me taking traditional medicine. He also recommended seeking the herbalist.” The family input is critical in cancer treatment decisions.

### 3.3. Perceived Benefit and Compatibility

Another frequently stated reason for visiting a traditional healer was the perceived benefit of traditional healing, including hoping for a cure. Traditional healers are alluring as the survivors feel they gain spiritually and physically from seeing one. Robiah reported she was spiritually healed from practising Islamic traditional healing: “I recite the zikir (Islamic chant) and read the Quran after prayer. It is healing in a spiritual way.” Rizman whose cancer had metastasized to the uterus and lung visited an Islamic healer. She claims a calming effect: “I have the treatment from Mr. X, I feel at peace, so calm and so relaxed.” The healer advised her to pray to God and read verses from the Quran that relates to healing. Rizman further elaborates “Ya, I know this when we pray to God, God helps us.”

There were informants who reported seeing healers just for physical comfort. Norzaini was one of them: “Sometimes I went (to see the healer) just to make my body feel good.” The physical improvement may be immediate, which further convinced them of the bomoh's healing capability. Sayun who was confident about traditional healing as she could see it work, rationalized:

“Because previously my lump was hard, after that it became very soft and I didn't feel the pricking pain (anymore). It (the lump) becomes cooler, not hot, not painful. I felt OK. While waiting for the hospital treatment, I went to see him again.”

Another factor that influences the informant to practice traditional healing was what they perceived it as compatible with Western medicine. Further, the bomoh may help them deal with the side effects of Western medicine. For Robiah, it had to do with side effects of chemotherapy:

When I was doing chemo, I felt like collapsing, very bad. I went to Y (a well known Islamic healing place). I felt much better the next time I did chemo. I used (drank) the (healing) water he gave me. I always drank three or four times (a day).

Some patients were assured by the traditional healers themselves of the compatibility. Sami, who among the informants survived breast cancer the longest, narrates:

She (the healer) said, don't worry, there's no interaction and side effect. You can still get the doctors medicine and take mine (herbs). You can do both. Don't worry and don't tell the doctor (whom Sami consults).

Sami's demised sister also visited the healer for her cancer. Although she realized that her sister succumbed to cancer despite taking the healer's herbs, Sami still saw the healer when she was diagnosed with cancer believing that it was complementary.

Seven informants reported seeking traditional healers to help them cope with cancer challenges and ensure a cure. Rizman claims that “I cannot eat, cannot do anything and cannot walk. I want to be completely cured.” The survivors interviewed reported seeking more than one healer in their search for cure. Roziah visited several: “My friend introduced me to another traditional medicine man in (name of town). Then I went for another one. …We have a very famous one here, (name of healer). He is famous in (town in Malaysia)”. Roziah consulted a traditional healer as far away as Indonesia hoping for a cancer cure.

Going to the healer also gives them hope of recovery. Sami reported, “Not that I don't see any change. …after that, I realized there was some change. Much later, there was hope. Because I think you should have to do two things, medical and traditional. You have two hopes.” Rashidah who shared similar beliefs also recommends trying complementary treatment: “You get the operation, you go for medication. And together with that you do the Islamic or other.” Desperate for a cure, the informant Aisan sought out the bomoh; “ I went to a herbal healer in (name of town). At that time I was in very critical condition.”

### 3.4. Healer Credibility

Evidence from our study shows that healer credibility also influences a cancer patient's choice for traditional healing as complementary treatment. Several informants reported that they selected healers who had reputations for healing cancer. Rizman saw an Islamic healer believing the healer can cure: “Those people that see (name of a famous Islamic healer) recovered.” Nowan who went to another well known healer echoed a similar belief: “Before this many people came to see him for treatment. There are some who are cured after taking the medicine.” Sami tried a quite well known traditional healer who recommends herbs to patients, reportedly based on her research.

Patients who perceived a healer credible bear with the challenge of seeing one. For example, Robiah drove almost 200 miles to consult with the famous Mr. XX healer when she had issues with her chemotherapy. Rizman was lucky to be able to consult the well known Islamic healer, Mr. X even though it was a long wait to get an appointment slot with Mr. X. Upon getting one, it was another long drive to finally get to see him.

### 3.5. Reservation with Western Practice and System Delay

Several informants reported turning to traditional healers as they had reservations about certain Western treatments. Nowan narrates her experience: “Yes, I confirmed (her cancer) first with the doctor. (Then I went to see a healer) because I was afraid. I am not confident with the operation.” The husband compounded the issue when he did not permit Nowan to proceed with the recommended operation:

“As for my husband he will not allow because they want to operate here, take (skin) from here (points to her thigh). Then both parts will be painful. So which one will heal? I asked the doctor, can I do the chemo first, and then operate? Doctor says, cannot, because my tumour is reaching nearly 6 cm. So the doctor was afraid that the thing will not stick. The doctor wants to do the easy job, simpler. The doctor wants to take the skin from here (thigh), and paste. That's what I didn't like.”

Traditional healer practices are patient friendly. Islamic healers read certain Quranic verses into plain water for patients to drink. For Robiah, the water was supplemented with betel leaf and its condiments:

“When I first went with my sister, it was only for the healing water. Then he made me betel leaves with nuts and lime, for me to apply (on her lump) for a few weeks. Then the lump appears to be gone.”

Patients who want to consult a doctor may have to wait for some time before they can see one or get a procedure done. The delay in the medical system made them seek traditional healers. Sayun rationalized that she consulted a bomoh while waiting for her turn for surgery “because the operation appointment was still a long time away.” Roziah reported a similar situation: “So, meanwhile before the operation I have lots of time. So, I went for traditional medicine.”

## 4. Discussion

Findings from the study will be discussed according to the motives identified: (1) recommendation from family and friends, (2) sanction from family, (3) perceived benefit and compatibility, (4) healer credibility and (5) reservation with Western medical system and system delay.

By culture, Malaysians are “collectivistic,” that is their identity is not determined by individual characteristics, but instead by the collectivity or group to which the person belongs [40]. Being “relationship oriented,” their lives are centred on family, village, country, and social group ties. This entails the mutual and reciprocal obligations they have for each other [40]. The collective and relationship culture is especially dominant among the native Malays. This study showed that when a person was challenged by cancer, they relied on information and support from their family and friends. Cancer patients rely on close interpersonal communication networks [41]. Such a communication mode is part of the collective, relationship-oriented Malay. Thus, as this study revealed, they are able to draw on their family and friends as sources of information and support.

From their experience, cancer patients know that for them to heal from cancer was also in their family and friends' best interest. The survivors knew that their family and friends meant well. With that assumption, the survivors took their family and friends' recommendation to see a traditional healer, even in cases where they themselves did not believe in traditional healing, as reported by Robiah. The interest for a loved one to be well made some family and friends go beyond mere recommendation. As in the case of Robiah and Aira, their friends were willing to take them to the traditional healers far from where they lived. The study suggests that well-intended family members and friends recommend cancer patients see healers based on what they perceived as healer credibility.

According to Islamic tradition, an action like a healing effort works better with the blessing from parents. Further, a Muslim daughter or son is supposed to abide by his/her parent's advice [42]. Thus when the parent recommends one to see a traditional healer, that suggestion is not taken lightly. This culture helps to explain why survivors went to a traditional healer upon a family member's suggestion, including extended family such as a father-in-law. This study's findings on the family and friend's role in influencing patients to see a traditional healer is consistent with the findings in studies by Razali and Yassin [1], and Hasan et al. [12]. In Malay culture, family involvement in decision making is essential. This finding is supported by earlier studies elsewhere and locally [43]. Cancer patients count on the family council and support. The push factor to consult a traditional healer was greater when the family not only recommended, but also sanctioned the visit. Sanction from family members has not been recorded in earlier studies.

This study also suggests that some patients believed in the ability of the healer to help them heal or even cure their cancer, sometimes within a short time frame. The healing can be spiritual or physical [22]. The perceived positive outcome from seeking a traditional healer in this study is congruent with Tovey et al. [19], Al-Adawi [20], and Freeman and Motsei's [32] work.

Some healers are so well known and their credibility is so high that people come from near and far to see them. The well-known healer that Rizman visited, for example, has recently opened an Islamic healing clinic to treat patients from all over Malaysia and the region. This study's finding on healers' credibility as a drawing factor is congruent with the work of Razali and Yassin [1], Heggenhougen [16] and Al-Adawi [20].

Patients have reservations about certain medical procedures which they believe will be painful, thus preferring to seek traditional healers. For example, in Nowan's case, her limited comprehension on the needed surgical procedure compounded by the fear to undergo surgery prompted her to go to a traditional healer. Fear and misconceptions about Western medicine were also observed by Taib et al. [17] and Leong et al. [21]. Furthermore, unlike certain cancer treatments such as chemotherapy which produce side effects, traditional healer practices are user friendly. It often only involves reading certain Quranic verses, drinking water blessed with prayers by the healer, or eating recommended herbs.

There are limited cancer resources and trained personnel in the country and these limited resources contribute to extended waiting time [3]. Cancer patients in this study talked about having to wait for some time before they can see a doctor, get test results, or get their treatments done. They make use of the waiting time for an appointment or scheduled procedure to consult with a traditional healer.

In this study traditional healing was chosen as complementary to Western medical treatment as cancer patients want to be cancer-free. They leave no stone unturned and resort to traditional healing as it is part of their culture. As these survivors also consult Western medicine, we term their extra effort for a cure by seeing the healers as “double insurance.” The practice of combining both Western and traditional healing for a cure was also reported by Moodley et al. [22], Tovey et al. [29], and Freeman and Motsei [32].

## 5. Conclusion and Implications

Evidence from this study suggests that a survivor's decision to see a healer is primarily motivated by the survivors themselves, to be cured and well. The survivor's decision is guided by what is perceived as beneficial or expedient given her direct experience with Western medical treatment including system delay, and/or what she observed or heard through recommendations from family and friends. Then, there are the roles of others, the caring family and friends who share a common good intention for the survivor's healing. With the best of intentions, they recommend the traditional healer, believing or assuming or hoping that the system works. The influence of family members is further enhanced with their sanction role. Such a role is critical in the Islamic culture where an effort will not be blessed without family—particularly parental and spousal sanction. In fact, according to Islam, unless it's in matters that otherwise will be detrimental to her, a wife needs her husband's permission to do certain things [44]. However, in the case of seeking healing which is essential, the wife need not get her husband's permission. But Muslim women sometimes misinterpret the religious guide, for example, by listening to the husband to not do the operation. This cultural practice has resulted in some blaming religious requirements as factors influencing patients' treatment-seeking behaviour. Further, many husbands inappropriately use Islam to justify their authority and dominance over their spouses [34]. The involvement of family and friends in cancer healing implies that educational interventions to assist those diagnosed with cancer ought to include these other parties too.

A main attraction of traditional healing is that it is patient-friendly and it relates to participants' cultural practices and belief systems. The role of culture in influencing patients towards seeking traditional healers is supported by Moodley et al. [22], Struthers and Eschiti [23], Salan and Maretzki [25], and Chen's [26] research. Cancer patients who seek Western medicine could benefit from a more culturally appropriate health system. For example, rather than limiting family visits to a few hours a day, hospitals might allow extended, even round-the-clock family visitation.

In the case of cancer survivors who seek both Western and traditional treatment, the traditional healing can be seen as complementary to Western medicine, a stance supported by numerous studies including Moodley et al. [22], Malik et al. [24], Tovey et al. [29], Lerner and Kennedy [31], Freeman and Motsei [32], Moodley and Sutherland [33], and Kamil and Khoo, 2006 [45]. From this perspective, the use of traditional healing as complimentary treatment has its potential. In fact, an inquiry on the collaborative role of traditional healers and Western medicine shows that such strategic collaboration would benefit cancer patients [46]. The issue for Malaysia is that although an initial effort has been taken to implement TCM at public hospitals [11], currently traditional healing is yet sanctioned by the government health system.

Traditional healing is embedded in Malay culture [34, 35]. Whether it is endorsed by Western medicine or not, it is here to stay. A major concern of one affected by cancer is to survive the challenge and continue to live a quality life. As a fighter, one will try various means to survive. To quote a survivor whose cancer has recently recurred and who is desperately seeking healing ways which include Islamic healing, “There are various healing potentials. Just try….”

## Figures and Tables

**Table 1:** Profile of Participants.

No	Name*	Age	Highest education	Employment	Type of traditional healer	Years survived breast cancer
1	Nowan	38	Year 6	Sales person	Use herbs	9
2	Sami	61	Ph.D.	Lecturer	Use herbs	12
3	Aira	41	Master degree	Formally Bank manager	Use an egg	10
4	Sayun	35	High school	Factory worker	Use herbs	2
5	Rashidah	49	Diploma	Businesswoman	Use white turmeric	3
6	Roziah	56	Master degree	Government Officer	Use an egg, herbs & prayer	3
7	Robiah	46	High school	Home maker	Use herbs	9
8	Aisan	45	Bachelor degree	Government Officer	Use herbs	7
9	Nakiah	57	Nursing certificate	Retired nurse	Quranic healing	2
10	Norzaini	34	Bachelor degree	Manager	Use herbs & vitamins	4
11	Rizman	75	Ph.D.	Lecturer	Quranic healing	10

*All the names are pseudonyms.
